# Twelfth Annual Meeting of the Mississippi Valley Association of Dental Surgeons

**Published:** 1856

**Authors:** 


TWELFTH ANNUAL MEETING OF THE

### Mississippi Valley Association of Dental Surgeons.

The annual meeting of this association commenced its session in the hall of the Dental College, on Thursday, the 20th day of February, 1856.

The President, Dr. A. S. Talbert, of Lexington, Ky., being absent, the Vice President, Dr. J. G. Hamill, of Xenia, Ohio, took the Chair.

The meeting was called to order, and a letter was read from Dr. Talbert, expressing regrets that he could not be present, stating as cause the severe illness of his wife.

The Secretary, Dr. A. M. Leslie, read the minutes of the last year's meeting, which were adopted.

The proceedings of adjourned meeting, held in May, 1855, were also read and adopted.

The following gentlemen, having applied for membership, after undergoing examination, were duly elected:

Dr. Jas. O. Martin, Franklin, Ind.
" Edmund Osmond, Ft. Jefferson, Ohio.
" Geo. W. Keely, Oxford, Ohio.
" J. B. Newbrough, Dayton, Ohio.
" E. Bray, Evansville, Ind.
" D. W. Harris, West Liberty, Ohio.
" Jos. Richardson, Cincinnati.

Dr. Leslie read Report.

Motion of Dr. Taft, invitation was extended to all members of Medical, as well as Dental profession, to be present, and participate in the discussions.

ORDER OF BUSINESS.

Executive Committee Reported the following:

1. Report of Executive Committee.
2. Report of Officers.
3. Report of Standing Committees.
4. Addresses and Essays.
5. Miscellaneous Business.

Under the head of Miscellaneous Business is:

1. Does Continuous Gum Work bear the Test of Experience?
2. What is the Best Method of Preparing Gold for Filling Teeth?
3. What is the Best Method of Drying Cavities for Filling Teeth?
4. Has Block Work any Advantages over Single Gum Teeth?
5. What is the Best Method of Separating Teeth Preparatory to Filling.
7. Is Gutta Percha Reliable as a Base for Artificial Teeth?
8. Can Artificial Teeth, Moulded on Porcelain Base, be worn with advantage?

8. What are the various Pathological conditions of inflamed Dentine, and What Circumstances Modify its Treatment?

10. What is the *Modus Operandi* of Arsenic, Chloride of Zinc, Nitrate of Silver, Creosote, Tannin, &c., when applied to Inflamed Dentine?

Reports of Treasurer and other officers read and adopted.

AFTERNOON SESSION.

Dr. Taft moved that the Constitution be so altered that the name of Examining Committee be changed to Committee on Membership.

Committee on Miscroscopic Observation reported progress, and were continued.

The Corresponding Secretary stated that he had no report to make.

Dr. Watt reported that three hundred copies of the Prize Essay had been printed and distributed for revision; that the copy is now correct and ready for printing.

Dr. Watt read an able and interesting report on Dental Progress, touching upon the use of Gutta Percha, Crystal Gold, Drying Cavities, Instruments and Teeth, the progress of the profession in Europe, &c., deprecating the use of amalgams, as being deleterious in effect and influence, remarking that some of the profession were advocating the use of an article, the *supposed improvement* of which consisted in the addition of another *base metal.*

Signed, GEO. WATT,
H. R. SMITH.

On motion of Dr. Taylor, report accepted and adopted.

Dr. Ulrey moved report be published in *Register.* Carried.

Letter from Dr. W. Storer Howe, stating that recent illness prevented him from delivering an address. On motion, continued over to next meeting.

Dr. H. R. Smith was now called upon to deliver an essay. Said he had prepared one, but thought it unfit. Several members urged its reading, and accordingly read an interesting paper on "Dental Education. of which the following is a synopsis:

The Doctor proceeded to make some appropriate remarks relating to "Dental Education," stating that the sad results of Dental operations were caused by ignorance: teeth broken off, and diseases of the mouth improperly treated. Even many physicians are not familiar with the anatomy of the parts. A mechanic is not a Dentist. Pupils, after a few months' study, go about the country, and unblushingly proclaim their ability to perform all operations, not knowing that a knowledge of physiology, pathology, chemistry, &c., are necessary. These teach and explain the cause and effect, point out the treatment and process of operating with profit and satisfaction. It is now necessary to be well qualified. No longer can the quack find public favor. There is no longer any excuse; the facilities for thorough education are abundant, having now three Dental schools, which rank with their sister colleges.

Several motions were made relative to printing the Prize Essay.

Dr. Bonsall thought best to provide funds to pay the expenses.

Here Dr. Taft exhibited the cost of printing in pamphlet or cloth; also the relative cost of stereotyping, &c.

Dr. Griffith thought (since learning the cost), best to proceed at once, at ten cents per copy for pamphlet, and twenty-five cents for cloth. Every Dentist would want from fifty to one hundred copies to give to patients.

Dr. Goddard only wanted to know where money was to come from?

After some further discussion and explanation, it was agreed that the price to *members* should be fixed at six cents for pamphlets, and a committee was appointed to solicit subscriptions.

3,000 copies were subscribed for; of which members took 2,000 copies; Toland and Thorne, 1,000 copies.

Dr. A. H. Bancroft was then unanimously elected a member of the Association.

The regular order being disposed of, the President announced the next business to be the 1st item of Miscellaneous Business, "Does Continuous Gum Woık bear the Test of Experience?"

Dr. Taylor did not intend to discuss this subject, as there were other objects that absorbed more of his attention, but was gratified in observing the steady improvement made in this work. Satisfied that the failures which had occurred were on account of imperfect manipulation, improvements in material and composition may yet be made—if it could be made with less shrinkage and more strength, would be better. The old custom was to rely upon the adhesion of material to plate. A few cases of this kind are still worn, but others failed. Remedied by backings, rims and chamber, filling materials in so as to give strength, arrange teeth and solder, then build the gum as high, and, giving the form wanted, filling up perfectly between the rims. Exercise more care in removing from furnace; will check if suddenly cooled. One-half of the operations of last year went through the furnace only once before enamelling. Some patients he would not put it up for —, not as strong as gold work, but as good as block work; single teeth not so apt to break. Some patients use more force in masticating than necessary, prefer gold; but some cases required either blocks or continuous gum, to restore contour of face; in such cases, prefers continuous gum.

Dr. Ulrey—Experience limited; used as much as other country Dentists. Some cases required it; in such, has had the work made by others; when sufficiently demanded, will procure furnace, &c.

Dr. Watt—Experience limited; believes it will stand; it fills a place in mechanical Dentistry long desired; preferable to blocks; think improvements will still go on; inferior to gold work, but will succeed.

Dr. Keely—Little experienced; two pieces, 1st made temporary, eighteen months ago, and gave satisfaction; second, worn three years, without difficulty in mastication, is pleasant, comfortable and sweet; cracked by a fall; otherwise as perfect as at first.

Dr. Bray—No experience, but likes appearance.

Dr. Taft—"Dr. Watt told all I know;" in present condition, doubt its practicability, except in favorable cases; but is sanguine it will succeed in time; objectionable at present, and would not adopt; put up several cases, and the majority have broken down; put up as firm as possible; if not alike solid in mouth, will ultimately check; the frame-work must be sufficiently strong, independent of body, so that no strain will come on body; otherwise it will check; would not use except when absorption is complete, and where the plate would rest with uniform solidity in the mouth; difficult to repair; if not blown up in furnace, will snap in places, even with extra care, in two-thirds of cases; rather mend half a dozen gold cases than one of this; it has peculiar odor, not pleasant.

Dr. Horton—Saw many operations; obtained the right before it was right; Allen taught to put up without stays; several pieces stood as long as could be worn, and remained in good preservation; a number three and a half years in use, nearly all good; a few failed; the Doctor here gave his experience in a peculiarly difficult case, for a very fastidious lady, which succeeded very well; on the whole, however, not favorably impressed; the great difficulty is in manipulation.

Dr. Goddard—Experience limited; put up probably one dozen sets; some did very well; others caused some trouble; recently saw a set perfect as ever; the mouth had changed, but patient pleased; not certain whether Hunter's or Allen's material, having used both; three sets recently seen, all doing well; making a set now; had some break; but that is not the fault of the work, but of him who makes it; instructs patients to be as careful as of a *gold watch;* the last set made for a lady who is very careful, and believes they will last her for thirty-five years to come, if she lives that long.

Dr. Griffith—Some experience; can't say how much; some of first cases are still in good condition; some of the last are fractured; but this is not the fault of the work, but the workman; ladies will tell stories to cover accidents; case of one tooth broken off, said to be done in eating bread; pecked with hammer fifteen minutes, to reach plates; believes from experience that it will succeed; a Case to do as soon as go home; when asked, always prefer Allen's style of work; the reasons have often been given; formerly had much difficulty in baking; now forms body and burns to biscuit, and then puts on Gum and bakes; believes it will succeed; thinks his failure in one or two instances was because Dr. Allen said it was not necessary to fit teeth accurately to plate; but finds it better to do so, and then form continuous linings; such cases always stand.

2. What is the Best Method of Preparing Gold for Filling Teeth?

Dr. Bonsall suggested that nothing be said but what is *new*.

Dr. Taylor—"Then I will be cut off entirely from debate!"

Dr. Bonsall had reference to this meeting, and to different members repeating the same thing.

Dr. Goddard—Nothing new: believes if gold is well and properly packed, it matters but little about the form; uses Foil in pellets; also much sponge gold.

Dr. Griffith uses Foil in strips and rolls.

Dr. Bonsall—Nothing new, except anneals all, as needed, crystal gold in ladle made for the purpose; throws flame of blowpipe on the gold; for foil, uses wire frame.

Dr. Smith—Nothing new; since last spring have annealed more than ever before.

Dr. Ulrey—Prepares foil by cutting into from four to six pieces to each sheet, annealing previous to use.

Dr. Watt—Believes in annealing; important to do that the *last* thing before filling.

Dr. Taft—Nothing new; thinks nothing has yet been said on the subject, members evidently not understanding the definition of question.

Dr. Smith—Reference was had to relative merits of foil and crystal gold.

Dr. Leslie prefers foil.

Dr. Taylor—In favor of drawing out discussion to cover the whole ground.

Dr. Griffith moved the question be considered to cover the whole ground of preparation of gold. Carried.

Dr. Goddard—Don't know which is best; would want to fill for President Pierce first! has failed with ribbons, but now uses pellets and blocks; in large cavities, uses crystal gold.

Dr. Ulrey—Nothing more; but believes can make as good filling with foil as crystal; no experience in manufacturing.

Dr. Keely—Gives preference to Crystal gold in large crown cavities; makes better plugs, and, if found to be as pure as Foil, will be used in preference.

Dr. Richardson—Never used any but Foil; satisfied there is none better; think the difference of opinion like the continuous gum work, results from want of experience.

Dr. Taft—The two names," crystal" and "sponge gold," have been confounded; should be distinct; don't say which material is best; makes good fillings of Foil, and also of Crystal; sponge is gold in minute division, without definite form; crystal is in regular, definite form: prefers foil to sponge; sponge absorbs moisture more than foil; prefers definite crystals to either; good fillings depend upon *perfection of manipulation;* many fillings can be made of crystal that could not be made with foil; foil depends upon the walls of cavity for support; will fall to pieces if removed; must *anneal* in every case; lays on charcoal, and throws on a large flame with blowpipe.

Dr. Goddard—Heats in the blaze of spirit lamp; think the blast from blowpipe hardens the gold; some cases crystals are best, and others foil; refers to Badger's plan in last number of *Journal*.

Dr. Leslie complained of severe headache, and other indisposition, which was increased by discussion; has several points to advance, but can not do so now; had used but little crystal; best be slow in adopting new things; tried some successfully, but faith strong in foil; must press on sides of cavity with *direct* force; crystals had failed in excellent hands, and succeeded in others; prefers pellets, generally of round form; annealing would not restore adhesiveness *some* Foil, because it never possessed any.

Dr. Watt made some explanations.

Dr. Horton—Experience in sponge or crystal small; his instruments are imperfect, but had been successful; careful in making cavities, used pointed instruments, pressing gold well to the walls; made some splendid fillings; thinks he would like it if he had the necessary instruments; attempts at annealing had been unsuccessful; made gold harder and more unmanageable than before; would like to learn more about it; in danger of flooding, makes cavity "well-shaped," so as to fill rapidly.

Dr. James—Never annealed until this winter; first annealed by explosive flame, which worked well; next threw on a concentrated flame; this could not be worked at all.

Dr. Griffith—From what has been said, one would be lead to believe that cavities could be formed just as you pleased; had been unfortunate in finding many cavities in angles, right angles and stars; such cases roll gold into a ball, place in cavity and fill all parts, then with very fine instrument fill into the points; never could fill satisfactorily, unless could fill all parts of cavity full; seven years ago, put twenty fillings into one mouth, three of which were of the above described kind; a few days ago saw the patient, and found all perfect; if could always find round cavities, there would be no trouble; but with *points* it is more difficult.

Adjourned to eight o'clock to-morrow morning.

THURSDAY MORNING.

Society met pursuant to adjournment.

Yesterday's minutes read and adopted.

No. 3 of Miscellaneous Business:

What is the Best method of *drying cavities* previous to filling?

Dr. Taylor—Habit to first wash cavity; sometimes with alcohol, or when decay is extensive, a little Creasote; use cotton first and follow with tissue paper; paper not so convenient, but better than tton.

Dr. Bonsall—Nothing more than Taylor has expressed; is well pleased with paper.

Dr. Smith—Had used some paper; took the inside leaves of *gold foil books.*

Dr. Ulrey—Had used linen, but would use paper if it could be obtained.

Dr. Watt—Experimented with sundry things; used pellets of Buckskin; had progressed to "*warm air;*" thinks that best, as absolute dryness can be produced, as is shown by the whiteness of bone or dentine. The most convenient method is the air pump, (which was here exhibited and described). Care must be taken not to heat so much as to destroy the integrity of the substance. Having this instrument, would not need the tissue paper.

Dr. Bonsall—Cotton leaves a gloss on surface; paper a dull appearance, indicating greater dryness.

Dr. Watt—Hot air leaves it white; absolute dryness.

Dr. Taft—Used hot air pump much; leaves cavity perfectly dry. Often cavities get wet while filling; with this instrument, can dry perfectly; with cavity half full of saliva, wipe with cotton and apply heat, and make it as dry as before; with cotton could not reach the indentations; formerly in such cases removed all the filling and began anew, making fortifications better, now remove only the fortifications, dry out and proceed as before. It is important to have the cavity *perfectly dry;* this can not be done with cotton, linen or paper, but can by the hot air pump. An improvement has been made in this instrument by Dr. Osmond, which consists in filling the cylinder or chamber with fragments of brass or iron, or coils of wire, which breaks the force of the jet of air and retains the heat longer.

Many precautions are used to keep the cavity dry; generally direct patients to breathe through the nostril; sometimes crowd a napkin back so that breath can not reach the cavity. Some patients can't keep tongue or jaw still. Important to keep tongue still; if at work on any point, "up pops the tongue to see what is going on." Important to give directions to keep tongue, jaw and soft parts of mouth still. Some saliva, like oil, insinuates itself into every crevice; sometimes closes the *mouths of ducts* with blotting or tissue paper; but unless the patient will keep the tongue still, it is useless to put anything *there.* These and many other things must be resorted to, but must be governed by the case.

Dr. Griffith—Tried various things, but finds it depends more upon the tongue than anything else. Keep the tongue still, then either lint, paper or cotton can be applied to keep saliva out; generally uses only two broaches in drying, as takes less time; is careful to have everything ready in advance; requests patients to free the mouth from saliva; then wipes the mouth with the patient's pocket-handkerchief, dries cavity and proceeds to fill; prefers to use the patient's own handkerchief, then if it is not clean they can only blame themselves!

Dr. Goddard—First prepares cavity; has not as many napkins as Dr. Townsend, but generally about a gross, varying in size from 2 by 4 inches, to size of ordinary pocket-handkerchief; the most trouble in lower jaw; uses "*watch springs;*" rolls napkin smoothly around spring and places inside of the arch under the tongue; another outside of the teeth or between lips and teeth, then folds another napkin square, six or eight thicknesses, and places it over the tongue and directs the patient to hold it there; if patient can't hold it firmly, helps them. Sometimes places a spring covered with napkin in each cheek, this keeps the mouth open and cheek away from teeth; proceeds the same in upper jaw, except don't require spring and napkin under tongue; dries cavity as usual and proceeds to fill; if flooded, dries with cotton and paper, and begins anew. If saliva flows excessively, turns patient's head one side and lets it run out, so that he can keep cavity dry; don't care if lady wets herself all over! Uses silver or gold shield for drill, so as not to hurt corners of mouth.

Dr. Osmond—Suggests that in cases of excessive flow of saliva, blotting paper be placed over the ducts, then use springs and napkins as described by Dr. Goddard, the paper will effectually close the mouths of glands and the spring and napkin will prevent the paper from being removed by the tongue or otherwise.

Dr. Horton—Prepares everything in advance; uses a great many napkins and has them always within reach; directs patient to cleanse the mouth and sometimes washes the mouth with cold water; sometimes wipes cavity first with cotton wet with camphor, then dry and proceed to fill; if flooded, takes all out and begins anew, doing the work all over.

Dr. Hamlen—When unsuccessful in getting cavity dry with cotton, cuts or scrapes it dry with an excavator.

Dr. Taylor—Don't go the mechanical so strong as Dr. Goddard; puts only the corner of napkin in the mouth; napkins should be made of linen; never used the hot air pump; uses speculum with flange to cover tongue, and keep it from running about; much depends on keeping cavity dry; lets patient and sometimes a friend hold speculum; wants both hands free; important to commence and proceed right; when cavity is dry, have everything ready in advance; impossible to fill with strips and keep dry; have blocks of gold prepared in advance; often uses blocks large enough to fill the entire cavity; thus do it quick; the work is done out of the mouth; would refer to Dr. Badger's article.

Dr. Bonsall—Has found the fine birds-eye linen the best.

Dr. Goddard—Prefers damask table-cloth linen; has used speculum and suggested to Dr. Taylor, last year, a flange to cover tongue, but has abandoned it; patients get careless and let it twist into every shape; folds napkin and makes patient hold it.

Dr. Griffith—Nothing has been said how, certain teeth are to be kept dry; some cavities and mouths are very difficult to keep dry, especially what might be called the "*ground squirrel mouth,*" posterior and lateral cavities far back and low down; impossible to get such dry; apply cotton, and before can make second application, cavity will be full.

Dr. Taylor—Never saw a mouth without some latitude; first covers the mouths of ducts to prevent the descent of saliva; applies tongue holder with napkin to keep tongue down; don't extend the mouth any more than is necessary.

Dr. Goddard—Don't extend the mouth, but proceeds from point to point, as before described.

Dr. Griffith—There is no room for such arrangement.

Dr. Goddard—You open the mouthtoo wide.

Dr. Griffith—But there is No *mouth* to open.

Dr. Goddard—If there is no mouth there can be no teeth.

Dr. Horton—Had a case on labial surface of lower molar; failed at first and removed filling, but afterwards succeeded in keeping dry and filling.

4th. Has block work any advantages over single teeth, not counterbalanced by disadvantages?

Dr. Goddard—Little experience; one case obliged to use blocks; could get no single teeth to do; generally uses single teeth.

Dr. Bonsall—Uses single teeth, except in peculiar cases where blocks are necessary to fill up; prefer single.

5th. What is the best method of separating teeth, preparatory to filling?

Dr. Griffith—Separates incisors with thin fine file; grasps with thumb and middle finger, with fore finger resting on the end inside of mouth, so as to give the file a slight curve; trims edges with smooth file; rarely ever uses wedges; if decayed so as to require filing, edges thin and ragged and would have to use file; finds inflamation of dentine to proceed from wedging; ordinarily uses nothing but file; but is governed by the case, and uses chisel when he can.

Dr. Bonsall—Uses wedges to separate so as to ascertain condition; dislikes to mar the symmetry of mouth of a fine young lady or woman.

Dr. Goddard—Here remarked that he was indebted to Dr. J. D. White for the idea of using watch-springs in the mouth, as before described: for separating, uses chisels of various shapes and sizes; opens all separations with chisels, wedges, and India rubber; young patients, particularly, uses rubber wedges, etc.; the Bicuspids and molars file freely of a V shape; uses file after filling, to trim up edges; uses file as little as possible; file both teeth if at all.

Dr. Bray—Practiced with wedge and file; generally with file; young ladies' and children's teeth are easily separated by wedging, and in order not to disfigure, uses wedges, except in back teeth; there files freely.

Dr. Horton—Prefers file; often uses chisels of select shapes; uses fine and smooth file; files both before and after filling.

Dr. Hamlen—Uses files and wedges about equally.

Dr. James—The advantage of cutting instruments over files; in sensitive teeth—there is less jarring and pain.

Dr. Ulrey—Uses chisels, and finishes edges with file; in large separations, can do all with file as well as chisel, except when sensitive; chisel produces less pain; as a general rule, thinks file best; but many cases of young persons would wedge, also older patients when borders are strong.

Dr. Goddard—It is generally understood that the file is used, but is it best? Would ask Dr. Ulrey if with an approximal cavity with ragged edges, would he or any other member separate with a file? First excavates. then fills the cavity full, then files tooth and gold together.

Dr. Griffith — Had cases with approximal cavities, and used wedges and India rubber, but always uses a fine file in front teeth.

Dr. Goddard—Had separated with wedges for lady fifty-five years old, who thought there was decay between, but found on examination all sound.

Dr. Ulrey—Desires to know what, if any, Bad results from wedge?

Dr. Griffith—Saw one case; lady had been ill several days; there was much inflammation; teeth had been wedged; inserts wedge but little at first, and increases daily; heard of much complaint and suffering from wedging, and seen some bad effects; may be fault of patient, but prefer file; always used file; now fifty-five years old, and if should live to be a hundred, would continue to file.

Dr. Bonsall—Thinks no injury can result from wedging; has tried many cases; in some kept it up for a month without ill effect.

Dr. Griffith—The peculiarities of ill effects of wedging—teeth are sore to the touch; this, after wedges had been out several days; it is delicate point to know how far to go, some can bear more than others; if all could endure alike, there would be no difficulty; the gums were healthy; no supuration; used red cedar for wedges; great faith in file.

Dr. Keely—Has great faith in file; thinks many failures attributable to the file or chisel not having been used; edges of wall must be strong; wedging makes teeth tender and sensitive, so that it hurts too much to fill properly; first files, then wedges close to gum; next day wedges again, so as to get through as soon as possible; patients will stand to be *hurt badly for short* time,better than a little for long time.

Dr. Ulrey—Much has been said of cutting and wedging; our friend from Oxford has much inflammation; no wonder, he hastens the operation too much; the more rapid the separation (by wedging) the greater the inflammation; better take more time, a week or more; instruct the patients how wide a space is wanted, and let them tighten a little every day, and then let rest a few days before filling; dislikes to cut away and disfigure teeth; tries, when can, to get along with wedging, except when the edges are very thin.

Dr. Hamill—There are cases, especially teeth with very small necks, and in young persons, where use wedges; trim with file after filling; can't see how injury could result, except by too much haste; forcing the tooth more rapidly than the alveolus, against which it is pressed, will absorb; force should be gradual. In the case mentioned by Dr. Griffith, the pressure was on the whole arch, the teeth resting against each other, and of course inflammation ensued.

Dr. Keely—Finds it necessary to be controlled by circumstances; some patients would remove wedge as soon as out of sight; can not control patients; finds no difficulty either with the patient or in the effect when the operation is hastened; uses wedges close to the gum; would not condemn the file, nor yet use it indiscriminately.

Dr. Horton—Had such a case as mentioned by Dr. Hamill; the patient had been to another dentist, and would not permit him to separate; fillings in a few weeks came out; when came to me, filed and filled, and doing well; would file thoroughly; where teeth have large necks, leave a shoulder to prevent them from coming together.

Dr. Ramsey—Uses cotton pressed gently at first, gradually increasing the force for two or three days; directs patients how wide space is wanted, and lets them increase the force from day to day; occasionally file; used wedges, but prefers cotton; cutting instruments are better than file, where they can be used to advantage.

Dr. Horton here inquired of Dr. Griffith how he kept files clear, whether with water or napkin?

Dr. Griffith—Dip file into water every few strokes, and wipe frequently; the question is really amusing; uses warm water in winter; would mention what might be advantageous to young men; as an invariable rule, would not be directed or influenced by patient; if the President were to take his chair and attempt to direct how the operation should be performed, would say, "Sir, if you have not confidence in me as an operator, I can not work for you at all." The doctor related a circumstance of the kind where a certain judge attempted to instruct how to perform operation.

Dr. Ulrey here requested Dr. Horton to make some explanations.

Dr. Horton—No cases have come under observation where thought wedges necessary; does not cut to disfigure; never resorted to either wedges or cotton; has instruments adapted, and may use if cases requiring it are presented.

Dr. Leslie—Experience tends to the use of the file or cutting instruments; some cases would not feel justified in cutting as much as would be needed for separation; then would wedge; if filed, should be so that edges would not come together; in small cavities, cuts cavity out entirely; some teeth the crowns are much larger than necks; here would feel justified in wedging.

Dr. Taft—Governed by circumstances; separate some by pressure, when space can be obtained, constitution good, and teeth will bear it; sometimes use both pressure and cutting; must have sufficient thickness of edge to hold filling; where teeth stand close, and no room to cut them, then cut from all the teeth to be filled; when much is to be removed, use cutting instruments; the operation is more rapid and less unpleasant to patient; no matter how much dentine is inflamed, can use cutters; in cases of perostitis, can use chisel; would not dare to use file; file motion in opposite range with axis of tooth; pressure from side to side; the force of chisel in a line with axis, and always in same direction; there are from fifteen to thirty cutting edges to file, only one edge to chisel; use file after chisel, first coarse then smooth; never would use file to separate; can do it in a half minute with chisel, where it would require from five to ten minutes with file; chisel is particularly applicable to the posterior surface of molars; one advantage over file is, that they touch only the tooth under operation.

Dr. Griffith—Asks if small decay in approximal surface of incisors would be left until it admits of filling and separating with pressure; or should file be used to cut decay away, he now says and wants it known, that he would take a fine file (must be fine); would take fifteen minutes if necessary; much injury done with coarse files; after filing, invariably dresses with Arkansas oil-stone; would pursue this course if the last he had to do.

Dr. Ulrey—In thick edges, small holes—would cut out, but when the cavity is deeper, if separated with file would cut one-third of tooth away and very much disfigure; would separate with wedges, fill, and allow them to return to their places. Some speak of leaving shoulders; there are many cases where this cannot be done.

Dr. Taylor—Some cases prefer wedging; hardly ever wedge except when filing is necessary afterwards; if edges are allowed to return together, decay will proceed; always takes precaution to cut away palatine edge most; when can be done effectually, prefers wedges; sometimes takes out a bicuspid to make room for others to separate. As Dr. Ulrey has just remarked, it is generally impracticable to leave shoulders; in bicuspids prefers making separation and leave necks resting together; this however is not always practicable. In cases of small necks, cuts away on inside sufficient for reception of drill to free cavity of decay; the tongue will keep the parts clean; instructs patients to use floss silk or pass handkerchief between. Where bicuspid and molar rest together, both decayed between and *bicuspid* much decayed, cuts *it* away and polishes surface without filling, and thus gets a fair chance to fill molar; if both are filled, will come together and decay.

Dr. Watt—Read report of committee on cabinet; efforts have been made to fill it up; and recommended that it should be completed. Report received and placed on record.

Adjourned until 2 o'clock this afternoon.

AFTERNOON SESSION.

Minutes of forenoon read and adopted.

The subject of the best means of preserving the color of natural teeth requiring filling, was discussed at some length.

Association then proceeded to the election of officers for the ensuing year. The following gentlemen were elected:

*President*—Dr. J. B. Newbrough.

*Vice President*—Dr. E. Bray.

*Secretary*—George Watt.

*Treasurer*—C. Bonsall.

*Executive Committee*—Drs. Taft, Ulry, and Richardson.

*Committee on Membership*—Drs. Taylor, Smith, and Taft.

*Committee on Dental Progress*—Drs. Watt, Smith, and Taft.

Drs. Osmond, Keely, and Bray were appointed to read essays at the next meeting.

Miscellaneous business was then brought up for discussion.

Item 7th, Is gutta percha reliable as a base for artificial teeth?

Dr. Taylor stated that gutta percha had been recommended only for temporary work, and for that he believed it to be reliable. Had used it pretty extensively for two or three months; in all cases except one had answered the purposes for which it was recommended as well as could be desired. One case appeared to fail; in this case but very little gum could be applied without making it bungling. When the teeth are recently extracted, there is insufficient room to apply. There are cases of partial sets where several teeth remain; had found great difficulty with metallic plates; in these gutta percha is better than anything else; prefers gold to anything else; put in case three months ago; had much trouble from gold plate; simply when teeth came on ridge of lower border; patient now wears gutta percha with comfort, having all the suction that is wanted; had another set to make by recommendation of the patient; have made one or two sets that might be called permanent; one objection—it is bungling in the mouth; another is, that after worn and much strained, it yields around the necks of the teeth. In constructing, plate must be heated as well as gutta percha before being applied. The teeth should also be warmed; then when cold will contract close to plate and around the necks of the teeth. Heat of food, etc. not sufficient to affect it; there is one difficulty, which is, that it can not be polished. Tobacco stains and colors it; one case, an inveterate chewer, still it faded but very little. Regards gutta percha, continuous gum work, etc., as additions to the means of practice, filling voids heretofore severely felt; deems it now only in its initiatory—improvements will go on to make it harder and better.

Dr. Goddard—Had used gutta percha and liked it exceedingly; several cases now on hand; one case at home; having used gold, likes gutta percha better; eats well and comfortably; clean and pleasant; believes it to be in its infancy, and hopes to see it improved to be just what is wanted.

Dr. Bonsall—One case put in last Saturday; the teeth were extracted on last Thursday; patient called to-day to have pressure on particular points removed; said if the question came up in Society to give her experience, as it was all she could desire, and could eat with comfort.

Dr. Ulrey—Some fifteen or twenty months' experience; had used the gutta percha of commerce and failed, although had bleached it white; put up several cases of the new gutta percha, and thinks it is just what is wanted; don't know how it will stand, but is easily appiled or changed; alterations can be made in a few minutes; and willing to take it on trial, at least as it is the most easy and comfortable kind of plate work: thinks it will be improved, but even now looks more like meat than the glassy continuous gum work.

Dr. Taft—No experience, but has seen some; the ability to adapt to mouth is desirable; it yields easily, can be warmed and fitted in the mouth; seen one tooth broken out of a case; believes it to be firm enough.

Dr. Ulrey—One thing has been overlooked; the mode of applying; uses narrow strip of silver plate. Backing as usual; using the teeth made for continuous gum work, leaving spaces between; then fills in front and back; prefers to fill in with finger on the cast; dips finger into hot water, trims with knife and smooths up; if not a perfect fit, warms, fits and trims further in the mouth. It is necessary to be kept perfectly clean.

Dr. Bonsall—Very important to keep clean.

Dr. Taylor—In manipulating, clean hands and clean sand, and everything clean, are necessary. Get up model and fit same as gold, but not by swaging. Cut out what is wanted, and stick the model in sand; make metal die and fit the rim; arrange the teeth: stick with wax and try in the mouth; take out and file down the silver plate as narrow as possible; don't want it wider than base of teeth; the narrower the better it can be adapted; then take it back to the model; all dry and clean, set on the plate warm, and build up with scraps warmed in flame of spirit lamp; both surfaces must be dry and warm to make them adhere.

Failures are occasioned by imperfect working and manipulation; if it does not fit the mouth, warm it, and with the fingers hold it to its place until it sets; any imperfections can thus be remedied; a case not fitting at all, may be fitted to the mouth and perfect suction secured. Common plate teeth won't do at all; continuous gum teeth or teeth made expressly must be used. Silver is as good as gold; no polish is required to metal. Back and strengthen same as other work; have made chambers, but just as good without. Gutta percha, when warm, has enough elasticity to yield when bite on one side, and yet not tilt or fall. Can be easily adapted, and then sticks like wax; there are many cases where it can not be used, but wherever there is sufficient absorption to require gum teeth, there gutta percha can be used.

Dr. Ulrey—In cases where teeth are short, lets the silver plate rest on gums, covering and extending the gutta percha, thus overcoming some of the difficulties.

8th. Can artificial teeth, when moulded on a porcelain base, be worn with advantage?

Dr. Taylor—No experience, but seen enough to believe the question may be answered in the affirmative; had sent to Dr. Saterthwaite a model of lead, and received in a short time an operation bearing the appearance of specimens on table; was surprised that the operation had not been ground; it fit and adhered to the mouth well; after four or five metalic plates had been tried unsuccessfully, seemed to have been moulded on cast and baked, gum applied afterwards. Could unhesitatingly answer question affirmatively; but whether it would come into general use, is another question.

Dr. Horton—(taking up the specimens). Had seen both Loomis' and Saterthwaite's work; one of these specimens is Loomis', the other Saterthwaite's; could answer question in the affirmative; "it can be worn to advantage in some, but not many cases." Living in neighborhood of Loomis and Wright of Cleveland, had opportunities to observe; can make no calculation for shrinkage of porcelain; experiment by sawing casts and afterward grinding to fit mouth. A patient of his had objected to metalic plate on account of taste in mouth; soft parts were very soft, and palate hard as rock; Loomis had tried nearly a month, but could not get suction; would not hold up one-half ounce and tilt by slight touch of finger; then opened correspondence with Saterthwaite; sent him wax impression and articulating model, and in a few days received a set of teeth not ground a particle, as was evident from small rough particles of enamel adhering to different parts; put it into the mouth, and would venture to assert that no man in this room, could, with thumb and finger pull it down; afterward ground off the rough particles with corundum slab, and now fits perfectly; can masticate well; eats *boarding house steak* without difficulty; now watching progress; whether it will stand the test of time, can't tell; washes like natural teeth; has observed no absorption.

Dr. Hamill was called upon for an address; he remarked that he was still young in the profession, but proposed to scare up the game and let others catch it; he then read an able and highly interesting essay on the various pathological conditions of inflamed dentine, which was ordered to be published.

EVENING SESSION.

Minutes of afternoon read and adopted.

Dr. Richard Peckover of Paris, Kentucky, and Dr. John F. Mercer, of Canada, were elected members of the association.

Dr. Taft, suggested that members and others meeting with subjects in practice upon which they would like to have discussion, should forward as early as practicable to the executive committee.

No. 9. What are the various pathological condition of inflamed dentine, and what circumstances modify its treatment?

Dr. Taylor—This subject elicited remarks from our previous President, as published in the January number of Dental Register, he here read from page 164; wish to draw attention to these facts; will view them as facts, and draw reasonings from analogy. Dr. Hamill's treaties in this connection, explains that there are numerous tubulæ folding upon themselves; some points are more sensitive than others; often more sensitive distant than near the nerve. First fact—in every carious tooth the pulp becomes impregnated by calcareous matter. As we approach pulp, we have increased sensation and more arterial blood; calcareous matter is supplied by the blood; occasionally find entire ossification of pulp; the calcareous matter becomes harder than the tooth itself; certain points on pulp. may be called islands of calcification appearing like original enamel basis; being a dense coherent mass; this is osteo dentine; is it insensible or like true dentine? Treatment—drill through edge and expose nerve; formerly, Risodontropy. There is more than one kind of caries; some soften rapidly; earthy portions should be removed, leaving the animal structure. Decomposition may be arrested; and in such cases better leave a covering and fill, than to destroy nerve. Treatment—uses both local means and constitutional remedies. Defer any further remarks for the next question.

Dr. Goddard—Not posted; don't think any one is except Taylor.

Dr. Hamill—This subject has already been laid over several years; better exchange opinions as far as can.

Dr. Griffith—Don't feel posted; but from some remarks of Taylor, will make a few remarks confined to points of experience. There are two characters of ossification of bone; one has character of new formation of bone, representing enamel as near as may be; the other where caries has been arrested, the whole character had changed, become hard and impervious to instrument. This should not be disturbed except to dress; as a protector where new bone is formed over nerve, *unless* there should be a fissure where acid may penetrate and produce inflammation, would leave it alone. Experience in many cases teaches that if gangrene or calcareous matter is soft remove it, but in ninty-nine cases out of one hundred it is hard and advisable to leave all or a portion; may thus fill to great advantage; filled many and heard of no ill results.

Dr. Richardson—In connection with calcareous deposite in pulp, much that is said is, perhaps, speculative; the deposite can only be accounted for by analogy. Teeth impaired by caries have less vitality; calcareous deposite is greater in old than young persons; accordingly, the disposition to deposite in proportion as vitality is imparied; don't know if pathologically increased sensibility at the junction between the dentine and enamel has ever been accounted for; the general habit has been to consider the teeth as distinct from other parts of the system.

In amputating a limb, the patient suffers most *under* the cuticle; beneath the enamel as beneath the cuticle, there is increased sensibility, because closely interlaced with nerves. This may be old to others, but if there is any other explanation, would like to hear it.

Dr. Taft—Regret we are so ignorant; feel the want of knowledge; there are some facts at which we can arrive, and from thence reason to others; pathology to physicians not of more importance, can not treat and arrest caries without a knowledge of the general organization; teeth sympathise and change with the rest of the system; if the general system is in an inflammatory condition, the teeth partake of it; if febrile, they also partake; many variations in organs, which can not take place in dentine; but is partaken of, being ramified by nerves, etc. When there is inflammatory diathesis and general system febrile, the teeth partake of it; sometimes affected by slight agents, and sometimes resists active ones. Destruction of dentine often takes place without inflammation; there are many modifying circumstances. Teeth often decay without apparent cause. The nature of dentine excludes any evidence of inflammation except exalted sensibility; sometimes find a point sensitive on walls of cavity, separate from pulp or the point of union between dentine and enamel; other times inflammation between dentine and enamel, at the termination of nerve ramification or redublication of nerve. Reason of exalted sensibility may be in greater irritation; may be in original defect of dentine; may be constitutional; many cases entire body of dentine inflamed; cut off at neck and often find sensibility; can not in such cases file up to fit pivot tooth; the nerve is more or less diseased; destroy nerve and all sensibility is gone; dentine may be sensitive at particular points or between the enamel and yet the nerve may be sound; when such is the condition it is not dependent on local, but constitutional means; antiphlogistic treatment will restore in short time; if filled without treatment, it kills the nerve; may use tonics, astringents, ect.; but escharotics do no good, especially arsenic.

Dr. Horton—Old men for council and young men for war. When attempt to fill and find opaque hard and can be scraped, there is no trouble; when diseased part is redish like liver, can cut with impunity; when a little lighter, seldom finds inflamation where sensitive between enamel and dentine.

Dr. Taft, remarked when nerve is destroyed inflammation of dentine is gone.

Dr. Horton—Destroyed nerve ten days ago, next day introduced excavator preparatory to filling, and patient jumped nearly out of chair. Opened out with drill; one side looked red and inflamed; with care, excavated and filled.

Dr. Taft—The nerve was not all removed; red globules can not pass through dentine. Nerves that ramify dentine, proceed from pulp.

Dr. Horton—Certain, removed all the nerve; not sensitive evening before, but sensitive next day.

Dr. Taylor—Had a case for treatment; patient said nerve was exposed; on examination, found nerve gone; imagination lead patient to believe it hurt, and would jump whenever toŭched; finally extended instrument to end of cavity, and, after many contradictions convinced patient that there was no nerve there; believe the man felt hurt as much as if the nerve had been touched; some people *will* be hurt; touch tooth with inflamed pereosteum, will jump as if nerve was touched; can't believe that any sensibility remains in tooth after pulp has been removed, any more than there would be in a finger, after separated from hand.

Dr. Horton—Can't be mistaken; patient perfectly cool.

Dr. Watt—It is a physiological fact, if a man loses an arm, he afterward feels pain in end of fingers; so with a leg, pain in toes, same as if they remained attached to body; had himself felt pain in a tooth after the nerve had been removed; removed nerve from young lady after tooth had been cut off, and all sensibility gone; next day introduced instrument; very sensitive; lady remarked, "Always had been very *nervous;* perhaps it had grown out again;" portion of nerve may remain after cut off, and pain will be felt; after some examination, removed a little "worm," which was, perhaps, a portion of nerve.

Dr. Ulrey—Is it not possible to cut off main nerve, and yet leave filliments and ramifications in the tooth, to leave life and sensibility? Teeth are highly vascular bodies, and full of life; filliments may remain protruding, and tooth be sensitive after nerve is removed; some patients won't flinch, whether they know or not that they will be hurt; inflamed dentine is to be treated, but how, except by poison?

Dr. Griffith—On questioning patients finds the generality are fond of acids, vinegar, acid fruits, etc.; when teeth are carious, acid acts rapidly on dentine; may remove pulp entire, but remains sensitive at the end where it joined the trunk nerve; acid acts on dentine; notwithstanding all nerve may be removed, soreness will be felt in cases where there is much inflammation of dentine; can not remove all inflammation from bony structure immediately; advises bi-carb. soda; next day, all soreness gone; nineteen times out of twenty, the little branches are destroyed in removing pulp; cut down a tree, and you destroy branches.

Dr. Joseph Taylor—When internal nerve is destroyed, in what direction, if any, does it communicate with main nerve? If nerve is destroyed, the connection is cut off; unless you can find a connection, there can be no pain communicated to brain; there may be soreness, but no pain.

Dr. Griffith—Is vitality destroyed by removing internal supply; there are *two* points; cut off internal, and yet external supply of blood will keep up vitality.

Dr. Joseph Taylor—The crown, as far as enamel extends, is supplied from inside.

Dr. James Taylor—Celebrated surgeons insist that the internal nerve, or pulp, is not always necessarily destroyed by the death of tooth; took out a tooth split open, and found living pulp; in another case took out two teeth fangs entirely absorbed; nerve dead in one, in the other found blood, (not pus); if this is not an anamoly, how could vitality be kept up? Red blood could not pass through bone, but pulp might have been kept vital from external connection; how long absorption had been going on, don't know; would advise all to observe.

10th. What is the *Modus Operandi* of Arsenic, Chloride of Zinc, Nitrate of Silver, Creosote, Tannin, etc., when applied to Inflamed Dentine?

Dr. Bonsall—Tannin is very different from all the others, being astringent, while the others are escharotic.

Dr. Ulrey—There is a difference in each; arsenic, chloride zinc, nitrate silver, are somewhat similar—are liable to be absorbed, and produce death of parts; arsenic, if allowed to remain long, will pass through dentine and destroy pulp; those are escharotics, and are neither more nor less than *poisons;* passing through dentine by the absorbants, leave no life.

Dr. Watt—Important to know the pathology, and also the action of remedies; otherwise would be like a party of old ladies discussing the merits of salves to cure every ill; the majority of these are escharotics, producing effect by decomposition; alkalies destroy fibrine by absorption of water; some escharotics operate by their affinity for water; some operate without decomposition; these produce loss of vitality, unless counteracted by vital action; there are two opposing forces, one by decomposition, to produce death of part —the other, to keep it living, and the result is, a chemico-vital action; divide escharotics into two classes; 1st class liable to be absorbed—the other not; these form compounds with albumen fibrine and gelatine, and to some extent are soluble under favorable circumstances. Arsenic will be absorbed; it forms compounds with these articles, which decompose rapidly; nitrate of silver and albumen form compound 85 parts, albumen, 15 parts, nitrate silver, being insoluble, and can not be absorbed, but is slowly decomposed, leaving deposit of oxid silver, where the acid leaves it; the nitric acid, in contact with dentine, is taken up by the phosphate of lime, and carb. of lime, and therefore is not absorbed; chloride zinc forms definite compounds with albumen, and undergoes decomposition, forming an eschar, and, therefore, inert; no similarity between this and arsenic may appear so at first, but not afterward; more like nitrate silver, and somewhat similar is ter-chloride of gold; decomposition takes place instantly—combining with lime and moisture; instead of vitality being destroyed, activity may be increased; tannin forms definite combinations with these, vital energy being increased; creosote forms definite combinations with albumen; try them out of the mouth, and you will find that these articles do form definite compounds, with albumen fibrine, etc. One thing is clear, there is a great difference of opinion as regards what escharotics are absorbed; mercurials and a few others are likely to be; others are not.

To. Dr. Richardson—Seldom used arsenic; never did to inflamed dentine—probably never will; absorption is so deep, can not excavate all that is decomposed.

Dr. Richardson—Case: applied arsenic, excavated and filled; after a time, tooth broke; a part of filling came out; removed balance of filling; found it adapted to the walls, but decomposition appeared to have gone further.

Dr. Watt—Thinks no *line* to which absorption would extend could be defined.

Dr. Taft—Can't tell any thing about the absorption of arsenic; one case extended through within twenty-four or forty-eight hours; either taken through the walls or absorbed by pulp; scarcely ever use it, and don't intend to, except to poison rats; more rapid in some cases than others; chloride zinc don't kill; goes to a certain extent, then stops; arsenic outstrips every other agent.

Dr. Taylor—Can not agree in all positions; improper to use arsenical preparations on inflamed dentine; if apply chloride zinc where the lamina is thin, it may penetrate to the nerve; would like to get something to take place of creosote; uses it more and more, relying, to great extent, on its antiseptic qualities.

Dr. Griffith—Have used arsenic, and expect to do so; don't profess to be a chemist; knowledge derived from experience; thinks those opposed, being chemists, predicate their remarks *theoretically*, upon the effects it *should* have; used in many cases; one patient, after several years, returned and said it was the best tooth in the head; there is an antidote for every poison; apply antidote, and it will overtake and overcome it; apply $\frac{1}{2}$ grain, and you destroy the tooth, but take $\frac{1}{80}$ or $\frac{1}{60}$ grain, with a little charcoal and creosote; this will kill, and often can take out nerve without the patient knowing it; if first application is not sufficient, repeat, but not next day; wait three or four days; to say that he had preserved one thousand teeth in this way would be within bounds; his experience is, that a very small portion may be applied to sensitive teeth, then excavate, and fill without complaint, in ninety-nine cases of a hundred.

Dr. Goddard—Here described a highly interesting case, which he was called upon by Dr. Gross to see and take model. A boy of nine years, with a fleshy substance growing in and protruding from the mouth, so as to have the appearance of a hog's snout. On examination could see no teeth, but a fissure where teeth ought to be; had been growing from infancy; rapidity of growth alarmed parents, and induced them to consent to an operation. Cut away all, until the naked bone was exposed, when a beautiful set of teeth was revealed. Full particulars of this case, with engravings from plaster models, will be published soon.

Several appropriations were made, and a number of orders, on Treasurer for various purposes, after which society adjourned to meet again on the 24th day of February, 1857.

We regret that we have not space to give a *verbatim* report, as we look upon this as a most auspicious meeting; the attendance was good; and an unusual amount of intelligence and interest manifested in the discussions. The profession in the West have, by their acts, proclaimed themselves out of the "leading-strings." The great number of important inventions and improvements places them in the front rank of the profession, and fit associates for the members of any other liberal profession. They desire to join hand and heart with their eastern brethren in the advancement of Dental Science; the progress they have made is a fitting guaranty of what they *can and will do*. J. T. T.

### PRIZE ESSAY.

THE *Prize Essay*, for which the Mississippi Valley Association of Dental Surgeons awarded a premium of ONE HUNDRED DOLLARS, is now ready for publication, and will be issued as soon as it can be printed.

This is one of the best works of the kind ever published, and is filled with useful and interesting information, in convenient form for popular reading. The object being to impress upon the public the importance of early attention to the teeth, and correct dental operations.

Every dentist should supply himself with one hundred or more copies to sell, or present to his patients.

| | |
|---|---|
| Retail price, in pamphlet form, | 15 cts. per copy. |
| " " cloth binding, - | 30 " " " |

A liberal discount to dentists and dealers.

Orders addressed to "Toland & Thorne," No. 38 West Fourth street, will receive prompt attention.

### BELLOWS-BLOWPIPE, AND LAMP.

The best Foot Bellows, and the best Soldering Lamp ever offered to the profession, are those *invented* and constructed by Dr. Van Emon, of this city. Many of our best dentists are familiar with their merits, and could not be induced to do without them. Nothing but their high price has kept them from general use. We have made arrangements to furnish these at about one-half the price heretofore charged.

The price will not exceed $12 for Bellows, and $3 for Lamp.

### DENTAL INSTRUMENTS.

Having made arrangements with Mr. H. R. Sherwood, whose fame as a Forceps and Dental Instrument Maker is known and acknowledged throughout the country, we will keep constantly on hand a full assortment of Forceps, and other instruments, of *every style, shape, and form*, and also manufacture to ORDER, any description of instruments that may be required; believing from the superiority of material, and skill of workmon employed, we will be enabled to give entire satisfaction.

Dentists ordering new patterns, or shapes, peculiarly their own, would do well to send drafts of points, with minute description of size, etc.

☞ Instruments re-polished, re-pointed, repaired, or altered at short notice.

### PREMIUM EXTRACTING INSTRUMENTS.

The Mississippi Valley Association of Dental Surgeons, at their meeting in February of last year, awarded and paid a premium of Fifty Dollars for a set of *Extracting Instruments* made by Mr. H. R. Sherwood, of Cincinnati. We will be pleased to furnish instruments of same kind, style, and quality, to those who may desire a *splendid set.*

We CHALLENGE competition in producing Forceps, which for beauty of design and finish, alegance and symmetry of shape, ease and comfort to the hand, and perfect adaptation to the teeth, will compare with those made by Mr. Sherwood.

### FORCEPS.

| | | | |
|---|---|---|---|
| Broad Fancy Handles, | octagon joints, | Jeweled. | |
| do. | do. | do. | without Jewels. |
| Plain Broad Handles, | | do. | Jeweled. |
| do. | do. | do. | without Jewels. |
| Plain Broad Handles, | oval joints. | | |

The above, of every variety, of shape and pattern, manufactured by H. R. SHERWOOD, J. D. CHEVALIER, and other popular makers, at the prices of the respective makers.

Also, common Forceps at 65c., 75c., $1 and $1 25 each.

### CHEVALIER'S FORCEPS AND INSTRUMENTS.

We are prepared to supply the profession with every kind and description of instruments, manufactured by Mr. J. D. Chevalier, for the same prices at which they are sold at his establishment in New York.

### TURNKEYS.

Straight Shaft.

Fox's Turnkey.

Clark's Turnkey.

English do.

### ELEVATORS.

Hook shaped.

Punch do.

Right and Left shaped.

Spoon shaped.

Push and Pull shaped.

Kentucky Hook shaped.

Screws, permanent and detached.

Besides every other variety of shape, with Ebony, Ivory or Pearl Handles.

### PLUGGING AND SCALING INSTRUMENTS.

Steel Handles. Ebony Handles.
Ivory do. Pearl do.
Every size of Handles or variety of shapes.

### LANCETS.

Ivory Handles. Steel Handles.
Pearl do. Ebony do.
Pocket Lancets, with one, two and three Blades.

### EXCAVATORS AND DRILLS,

With Steel, Ebony and Ivory Handles of every desirable size.

### HAND MIRRORS.

Pearl, with Silver Mounting. Pearl, with Gold Mounting and
do. Gold do. Setting.
These, of various sizes and patterns.
Also, Rosewood and Mahogany.

### MOUTH GLASSES.

Pearl, Gold mounted. Pearl, Silver Mounted.
Ivory. Rosewood.
German Silver Handles, with joint for pocket.
The above, in great variety of patterns and styles.

### PORCELAIN TEETH.

We have on hand and are receiving a large stock and splendid assortment of Teeth, from the following celebrated manufacturers:

Jones, White & McCurdy,
N. Y. Teeth Manufacturing Co.
Porcelain Manufacturing Co.
Orum & Armstrong's.

Our stock comprises the *finest selections*, of all the best manufacturers, which we are prepared to sell at the following *very low prices:*

| | | |
|---|---|---|
| Gum Front Teeth, each, | - - - - - | 20 cents. |
| Gum Mo. and Bi. Teeth, each, | - - - - | 20 " |
| Plain Front do. do. | - - - - | 10 " |
| do. M. and B. do. do. | - - - - | 10 " |
| Continuous Gum Teeth, do. | - - - - | 10 " |
| Teeth for Gutta Percha work, each, | - - - | 10 " |
| Pivot Teeth, do. | - - - | 10 " |

All at the above prices except Molars and Bicuspids *without Gums*, of Jones, White & McCurdy's manufacture, which are 12 cts. each.

A liberal discount to those purchasing in large quantities.

☞ Teeth carefully selected to casts, or according to samples and directions, without extra charge.

Block Teeth, Continuous Gum Teeth, or Single Teeth, mounted and finished on reasonable terms. Also—Teeth mounted on Gutta Percha Base.

### METALS.

Gold plate 18 carats fine per dwt., 90c.

Gold plate, 20 carats fine per dwt., $1.

| | | | |
|---|---|---|---|
| Gold Solder, | 10 | carats fine, | 50c. |
| do. | 14 | do. | 75c. |
| do. | 16 | do. | 85c. |
| do. | 18 | do. | 95c. |

Gold plate, alloyed with platinum, suitable for Backs and Clasps, 90 cts.

Gold Wire, Round and Half Round, same price as plate.

Gold Springs, a very superior article, from $1 25 to $3 per pair.

### SILVER PLATE SOLDER AND SPRINGS.

Gold and Silver Plate cut carefully to patterns at the above prices.

### PLATINA.

French Plate. French Wire.
English do. Remelted Plate.
Thin Plate in strips for Batteries.

☞ Pure Gold, for Soldering Platina.

### GOLD FOIL.

J. B. Dunlevy's, $28 per oz.
Jones, White & McCurdy's, $28 per oz.
James Leslie's, $28 per oz.
Ashmead & Hurlburt's, $28 per oz.
Chas. Abbey & Son's, $32 per oz.

### TIN FOIL.

Jones, White & McCurdy's chemically pure Tin Foil.
Kearsing's Staniel Foil.
Also, several other manufactures.

### HOT-AIR PUMPS,

As described and recommended by Drs. Taft and Watt.

### A. J. WATT & Co's. PATENT CRYSTALINE GOLD,

Nos. 1, 2, 3 and 4, in $\frac{1}{8}$ oz. boxes, at $4 per box. Price $30 per oz., when 1 oz. or more is taken.

### DENTAL WORKS,

We are prepared to supply all the Standard Books and Text Books on Dental Surgery.

Also, receive subscriptions for all Dental Periodicals and Journals published in the United States.

### OPERATING CHAIRS,

Of superior workmanship, and any desired pattern.

### DENTAL CASES.

Every size and pattern—furnished either empty or handsomely trimmed and fitted with Instruments.

### SPITTOONS AND SPITTOON FUNNELS.

### ROLLING MILLS AND FORGES.

Of all sizes, at lowest rates.

### SYRINGES.

Pure Silver. Britania, with silver tubes.

### BLOCK WORK.

Executed in the most beautiful and artistic style, to supply the loss sustained by absorption, accident, or disease, adapted to all the irregularities of the mouth, of natural, life-like shapes, shades, and proportion, in sections to suit the case, from a *single tooth to a full set.*

There are many cases where Blocks are indispensible to fill up and restore the natural *coutour* of the mouth. Persons ordering should be careful to send correct drafts and articulations; also, specimen of the *color* desired.

### SPECIMENS ON HAND.

Block Teeth, Continuous Gum Teeth, Gutta Percha Work, and all kinds of plate work, mounted *exclusively for the Profession*, on reasonable terms.

Combining with our own experience the services of a skillful practical Dentist, the profession may rely upon having orders filled without delay, and teeth sent with strict regard to description and the requirements of each case.

### MINERALS.

| | |
|---|---|
| Feldspar, | Rotten Stone, |
| Silex, | Rouge, |
| Kaolin, | Crocus, |
| Lake Superior Stone, | Cobalt, |
| Arkansas Stone, | Arsenic, |
| Scotch Stone, | Ox Gold, |
| Asbestos, | Ox Titanium. |

Body and Gum, and all materials for Continuous Gum Work.

### FILES.

Murphy's, Stub's, Earnest's, N. Y. Teeth Manufacturing Co.'s, and others, of all shapes and sizes used by Dentists.

### TOOTH POWDER BOXES.

Plain Wooden, Wooden with labels varnished, Glass and Earthenware with metalic tops,

TOOTH BRUSHES, in great variety.

### AMALGAMS, ETC.

New Amalgam, as recommended by Prof. Townsend and others, Evan's Amalgam, Precipitated Silver, Slayton's Gutta Percha Filling, pure distilled Mercury, pure Silver, pure Tin and Cadmium.

### MISCELLANEOUS.

Hot-Air Pumps.

Saliva. "

Plain and Spring Sockets.

Sockets with revolving head.

Burs, Drills, and Exc. for Sockets.

Spring Plugging Forceps.

Baxter's Plugging Forceps.

Bur Thimbles.

Pivot Wood.

Floss Silk.

Sheet Lead for patterns.

Porcelain Slabs.

" Mortars.

Tooth Polishers.

Saw Frames.

Corundum Slabs.

" Files,

Broaches.

Broach Carriers.

Knives or Spatulas for Gutta Percha.

Portable Head Rests.

Foil Shears.

Napkin or Tongue Holder.

Cheek Holders.

File Carriers. of Chevalier's, Smith's, and Kern's pattern.

Colored Glass Spittoon Funnels.

Tooth Brushes.

" Powder.

" Soap.

" Pastes.

Letheon or Washed Ether.

Chloroform.

Kreosote.

Chloride Zinc.

Morphine.

Tannin.

White Wax.

Yellow Wax.

Gum Mastic.

Gum Sandarac.

DENTAL SKULL, exhibiting second Dentition.

Do. do. with full permanent set.

### MECHANICAL DENTISTRY.

Chevalier's Lathe.

Mead's Lathe.

Jones, White & Co.'s Lathe.

Grinding Machine, with Band.

do. do. with Cogs.

Hawes' Molding Flask.

Locke's do.

Ladles, for melting Metal.

Self-acting Blowpipes.

Ingots, Metal and Stone.

Draw-plates.

Plate Guages.

Bench and Hand Vices.

Saw Frames and Saws.

Burs and Saws for Lathe.

Copper Pans for Acids.

Soldering Lamps.

Corundum Wheels.

Brush do.

Hand Brushes.

Impression Cups.

Blowpipes.

Plate Punches.

do. Benders.

do. Wire Nippers.

do. Scrapers and Burnishers.

Anvils and Beck Irons.

Pliers, round and flat.

Slide Tongs, and Pin Vices.

Plate Shears, straight and curved.

Hammers.

Hammer and File Handles.

Bow Drill Stocks.

Teeth Holders.

Tweezers and Solder Tongs.

Furnace and Muffle Tongs.

Furnaces, one story.

do. two do.

Muffles and Slides.

Hunter's Materials for Artificial Gums.

Allen's Silicious Compound.

do. Gum Enamel.

Articulators.

Calcd. Plaster.

Asbestos.

Zink.

Lead.

Bismuth.

Antimony.

White Lake Sand.

Molding Sand.

Polishing Powders.

### PREPARATIONS FOR THE TEETH.

ZERMAN'S ANTI-SCROBUTIC TOOTH WASH — Produces beautiful *white Teeth, healthy Gums*, and *fragrant breath*. Price, at retail, twenty-five cents.

DA COSTA'S CELEBRATED TOOTH WASH.—This is a highly popular article for cleansing and beautifying the Teeth, hardening the Gums, and purifying the breath.

WATTERS' GENUINE TOOTH SOAP.—This delicious article combines so many valuable qualities that it has become a standing favorite throughout the United States. Dentists and physicians prescribe it with the best results.

HARRISON'S Dental Soap.
do. do. Tablet.
do. Rose Tooth Paste.
do. do. Powder.
do. Ebony Tooth Powder.
do. Pearl do.
do. Tooth Cordial.

BAZIN'S Charcoal Tooth Paste.

JOHNSON'S Tooth Soap.

DR. CRANE'S ODONTOSMEGIA—For cleansing and preserving the Teeth.

ROGERS' ROSE DENTIFRICE.—A pleasant Dentifrice for preserving and Cleansing the Teeth, and purifying the breath.

### HANDLES.

We have on hand a a fine lot of Handles of

DR. C. W. BALLARD'S PATTERNS,

BOTH IVORY AND EBONY,

Which we are prepared to fill with *Blades* of any desired size and shape.

Also, Twist Handles, various sizes.

*Punch* and Plugger Handles of Ivory, Ebony and Walrus.

### IVORY FOR SALE.

